# A comprehensive review of deep learning-based approaches for drug–drug interaction prediction

**DOI:** 10.1093/bfgp/elae052

**Published:** 2025-02-23

**Authors:** Yan Xia, An Xiong, Zilong Zhang, Quan Zou, Feifei Cui

**Affiliations:** School of Computer Science and Technology, Hainan University, No. 58, Renmin Avenue, Haidian Island, Haikou, Hainan Province, 570228, China; School of Computer Science and Technology, Hainan University, No. 58, Renmin Avenue, Haidian Island, Haikou, Hainan Province, 570228, China; School of Computer Science and Technology, Hainan University, No. 58, Renmin Avenue, Haidian Island, Haikou, Hainan Province, 570228, China; Institute of Fundamental and Frontier Sciences, University of Electronic Science and Technology of China, No. 4, Section 2, Jianshe North Road, Chengdu, Sichuan Province, 610054, China; Yangtze Delta Region Institute (Quzhou), University of Electronic Science and Technology of China, No. 1, Chengdian Road, Kecheng District, Quzhou, Zhejiang Province, 324000, China; School of Computer Science and Technology, Hainan University, No. 58, Renmin Avenue, Haidian Island, Haikou, Hainan Province, 570228, China

**Keywords:** drug–drug interaction, deep learning, molecular representations, graph

## Abstract

Deep learning models have made significant progress in the biomedical field, particularly in the prediction of drug–drug interactions (DDIs). DDIs are pharmacodynamic reactions between two or more drugs in the body, which may lead to adverse effects and are of great significance for drug development and clinical research. However, predicting DDI through traditional clinical trials and experiments is not only costly but also time-consuming. When utilizing advanced Artificial Intelligence (AI) and deep learning techniques, both developers and users face multiple challenges, including the problem of acquiring and encoding data, as well as the difficulty of designing computational methods. In this paper, we review a variety of DDI prediction methods, including similarity-based, network-based, and integration-based approaches, to provide an up-to-date and easy-to-understand guide for researchers in different fields. Additionally, we provide an in-depth analysis of widely used molecular representations and a systematic exposition of the theoretical framework of models used to extract features from graph data.

## Introduction

In recent years, the incidence of complex diseases has continuously increased, especially among the elderly population. Conditions, such as hypertension, heart disease, and diabetes often co-occur, significantly impacting patients’ health and quality of life [1–3]. Faced with this critical situation, traditional single-drug therapy is no longer adequate to meet current treatment needs. Therefore, to control and treat these complex diseases more effectively, the strategy of combining multiple drugs has emerged, bringing new therapeutic hope to patients.

Polypharmacy, as the name suggests, is the simultaneous application of two or more drugs to synergize the treatment of a disease. This approach takes advantage of the complementary effects of various drugs, making the treatment more precise and effective and helping to better control the disease [4–6]. For example, in diabetes treatment, drug combinations can act simultaneously on different physiological pathways, which not only help to lower blood glucose levels, but also improve insulin sensitivity, thus achieving more comprehensive and effective control of diabetes mellitus [7]. However, combination medications may cause undesired drug interactions [8]. Such interactions can lead to altered therapeutic effects, increased adverse effects, and even pose a threat to the patient’s life [9, 10]. Usually, drug interactions can be categorized as synergistic, antagonistic, or non-responsive [11]. Therefore, accurate prediction of potential drug interactions is essential to improve synergistic effects and minimize unintended drug side effects.

Traditional methods, such as database queries and biological wet experiments, are labor-intensive, inefficient, and costly in evaluating the possible interactions of multiple drug combinations. Additionally, ensuring the reproducibility of the results is challenging [12–15]. However, with the rapid development of bioinformatics and computer science [16], we have witnessed an influx of computer-aided computational methods that have demonstrated impressive results in drug–drug interaction (DDI) prediction [17–21]. In this review, we systematically sort out the commonly used databases and drug molecule representation methods, which provide a solid foundation for DDI prediction. We then review the deep learning-based DDI prediction methods in recent years, which can be roughly categorized into three groups: similarity-based methods, network-based methods, and ensemble learning-based methods. We also introduce several commonly used prediction models. Finally, we offer an outlook on future research directions.

## Data sources

In this section, we briefly introduce three databases commonly used for DDI prediction: DrugBank, TWOSIDES and DDInter. Detailed information on the two datasets is shown in Table 1.

**Table 1:** Summary of the available databases

**Dataset**	**Drugs**	**DDIs**	**Types**	**URL**
**DrugBank**	1706	191 808	86	https://go.drugbank.com/
**TWOSIDES**	645	4 649 441	963	https://tatonettilab.org/resources/tatonettistm.html
**DDInter**	1833	236 834	–	http://ddinter.scbdd.com/

Note: “–” represents that the information is not given in the database.

### DrugBank

DrugBank is a drug information repository that contains detailed information on a large number of drugs, including various aspects of drug-related chemistry, pharmacology, biology, and clinical information [22]. As an important reference tool for drug research and development, DrugBank includes both marketed and experimental drugs. It also records the interactions between these drugs, providing extensive details on drug mechanisms, targets, pathways, and their molecular interactions. Until now, the DrugBank database has recorded 1706 drugs with 191 808 data points on drug interactions, categorized into 86 different types of DDIs. Each drug entry in DrugBank includes a wealth of information, such as chemical structure, mechanism of action, therapeutic indications, side effects, pharmacokinetics, and pharmacodynamics. Furthermore, DrugBank integrates data from various sources, including scientific literature, regulatory agencies, and pharmaceutical companies, ensuring that the information is accurate, up-to-date, and comprehensive.

### TWOSIDES

The TWOSIDES database specializes in collecting information on side effects caused by DDIs, with a particular focus on adverse reactions arising from individual drugs in drug pairs or from more complex drug combinations [23]. The database contains a total of 4 649 441 event records involving 645 different drugs and 963 interaction types, demonstrating the diversity and complexity of DDIs. Each event record includes detailed information such as the drugs involved, the type of interaction, the observed side effects, and the clinical context in which the interaction occurred. It is worth mentioning that in the TWOSIDES database, a pair of drugs may correspond to multiple types of interactions, providing scholars with a valuable opportunity to conduct multilabel classification studies for a deeper understanding of DDI mechanisms. Moreover, TWOSIDES integrates data from various sources, including clinical trials, case reports, and post-marketing surveillance, ensuring a comprehensive and robust dataset.

### D‌DInter

DDInter is a comprehensive, specialized, and open-access database dedicated to DDIs [24]. The database encompasses a wide range of interactions with detailed annotations for each DDI association. These annotations include descriptions of the mechanisms underlying the interactions, risk levels, management strategies, and alternative medications, all aimed at enhancing clinical decision-making and patient safety. DDInter contains a total of 236 834 DDI records, covering 1833 approved drugs. The database’s structure is designed to facilitate easy access and analysis, with each record meticulously documented to provide valuable insights for researchers and clinicians. This extensive resource supports a deeper understanding of drug interactions and helps in identifying safer therapeutic alternatives.

### Molecular representation

**Figure 1:**
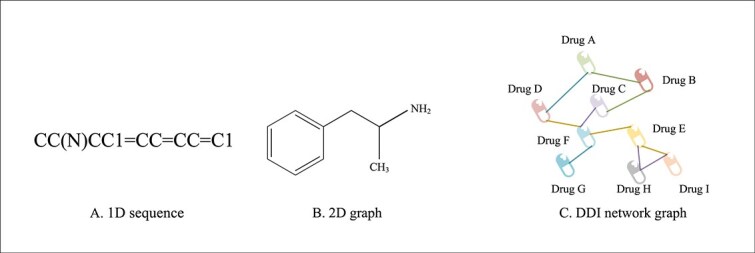
Illustration of the three common molecular representations for Amphetamine, including: 1D sequence, 2D graph, and DDI network graph.

The representation of drug molecules is a crucial step in performing drug-related tasks, such as predicting DDIs. For instance, amphetamine, a central nervous system stimulant and sympathomimetic agent, is commonly used for the treatment of attention deficit hyperactivity disorder. Its three common molecular representations are shown in Fig. 1.

### One-dimensional sequence

The SMILES (Simplified Molecular Input Line Entry System) [25] sequence is a linear code that represents a compound molecule. It explicitly describes the structure of the molecule using an American Standard Code for Information Interchange (ASCII) character string. It symbolizes the atoms, bonds, and other information in a molecule through specific naming rules and arranges them into a linear sequence of symbols in a certain order. A molecule can typically have multiple equally valid SMILES strings. For example, CCO, OCC, and C(O)C all represent the structure of ethanol. The process of generating a SMILES string for a given molecule depends on the normalization algorithm used, which generates a unique SMILES string for each structure. This unique string is known as the canonical SMILES. These algorithms first convert the SMILES into an internal representation of the molecular structure. Then, they examine the structure and generate a unique SMILES string. It has naming uniqueness and singularity, meaning that under the SMILES naming system, the name and structure of a molecule are synonymous. This means that whoever in the world uses SMILES to name a molecule will choose the same name to represent the same molecular structure. However, SMILES may have limitations in representing stereochemistry and complex molecules. Nevertheless, SMILES sequences have the advantage of low storage space requirements, making them a desirable method for representing chemical structures in computers.

### Two-dimensional graph

**Figure 2:**
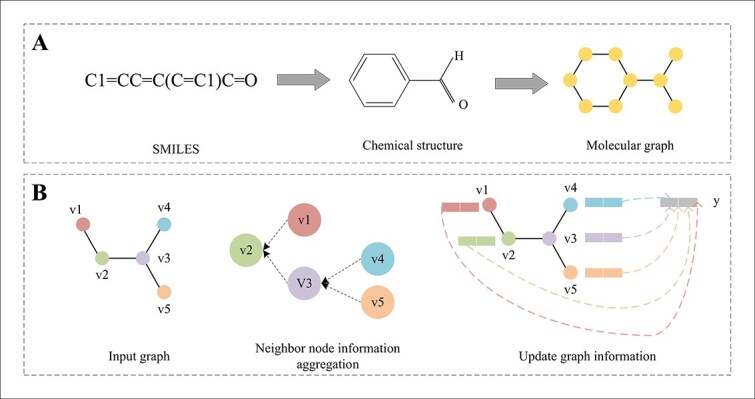
Molecule representation and graph embedding. (a) Preprocessed the smiles into graph. (b) The process of message passing: aggregation and updating.

Another more intuitive way of presenting drug molecules is by drawing two-dimensional (2D) graphs, also known as drug molecule graphs. It is common to use RDKit [26] to preprocess SMILES into a graph, where atoms are represented by nodes and chemical bonds are represented by edges, as shown in Fig. 2(a). Thus, the drug is denoted as $G=\left(V,E\right)$, where $V={\left\{{v}_i\right\}}_{i=1}^N$ is the set of nodes and $E={\left\{{\left({v}_i,{v}_j\right)}_s\right\}}_{s=1}^M$ is the set of edges. Each node ${v}_i$ corresponds to a feature vector ${x}_i\in{R}^{d_1}$ that describes its attributes and similarly, each edge ${e}_{ij}=\left({v}_i,{v}_j\right)$ possesses a feature vector ${x}_{ij}\in{R}^{d_2}$ that portrays a specific relationship between its connected nodes. The characteristics of commonly used atoms and bonds are given in Table 2. In contrast to sequence-based methods, graph-based representations are more intuitive and efficient in extracting structural information, which can be easily achieved through graph convolution operations.

**Table 2:** Atoms and bonds features

**Atom features**	**Description**	**Function**
Atom type	Atomic symbol	GetSymbol()
Degree	Number of neighboring atoms	GetDegree()
Formal charge	Formal charge of an atom	GetFormalCharge()
Hybridization	Hybridization mode of an atom	GetHybridization()
Radical electrons	Number of radical electrons	GetNumRadicalElectrons()
Implicit valence	Implicit valency of an atom	GetImplicitValence()
Aromatic	Whether or not the atom is aromatic	GetIsAromatic()
**Bond features**	**Description**	**Function**
Bond type	Type of bond	GetBondType()
Conjugated	Whether or not the bond is conjugated	GetIsConjugated()
Ring	Whether or not the bond is part of a ring	IsInRing()

### Drug–drug interaction network graph

To fully integrate and utilize the multidimensional information on the biology, chemistry, and phenotype of drugs, a drug–drug interaction network (DDI network) was constructed. In this network, drugs are abstracted as nodes, and DDIs serve as edges connecting these nodes. Thus, DDI prediction can be viewed as a network linkage prediction task aimed at accurately predicting and identifying new DDIs. A heterogeneous graph is a network graph based on heterogeneous data structures, consisting of different types of nodes and edges. Nodes represent different data entities and edges represent the association relationships between nodes. Heterogeneous graphs involve interactions between different types of nodes and edges, providing more information for model learning and thus improving the expressive and generalization capabilities of models. A knowledge graph is one type of heterogeneous graph.

### Technology of drug–drug interactions

The goal of the DDI prediction task is to construct a computational model that receives two drugs as inputs and outputs a result indicating whether or not there is an interaction between the two drugs. This task can be summarized as a binary classification problem aimed at determining whether interaction between two drugs occurs. Specifically, given a dataset of DDIs $S={\left\{{\left({d}_x,{d}_y\right)}_s\right\}}_{s=1}^C$, where ${d}_x$, ${d}_y$ are taken from the drug set $D$. The DDI prediction problem centers on learning a prediction $f:D\times D\to \left[0,1\right]$, which is designed to predict the probability of an interaction occurring between any two drugs. For more complex cases, i.e. determining the specific type of interaction that occurs between drugs, this multiclassification problem can be solved by transforming it into multiple dichotomous classification problems. Specifically, with two drugs and one interaction type as inputs, the model can be transformed into the $f:D\times D\times R\to \left[0,1\right]$. Currently, to effectively solve the DDI prediction problem, researchers usually adopt two main approaches: constructing drug similarity matrices and converting drug information into graph structures to extract features. These two methods have their advantages and can capture DDI information from different perspectives. Therefore, we systematically review the common models for constructing similarity matrices and extracting features from graph data, including graph attention networks and message-passing networks.

### Similarity matrix

Drugs have chemical features such as chemical substructures, targets, enzymes, and pathways of action, which provide a variety of information about the drug [27]. Each distinct feature is associated with a unique collection of descriptors, which can be effectively represented through binary feature vectors. All proteins, enzymes, and targets are listed in three separate vectors a transporter protein, enzyme, or target is relevant to the drug, that position in the vector is marked as “1”; otherwise, it is marked as “0”. By encoding these three types of information, a drug has three different vector representations. However, it is worth noting that these feature vectors tend to have high dimensionality and most of the coordinates in them have the value of 0, i.e*.* there is high sparsity. To process and analyze these data more efficiently, we usually employ feature compression to reduce their sparsity and improve computational efficiency. The Jaccard similarity function is a commonly used method. It helps us accurately calculate the similarity between pairs of drugs and also serves the purpose of dimensionality reduction [28]. The Jaccard similarity is calculated as follows:


(1) \begin{equation*} J\left(A,B\right)=\frac{\left|A\cap B\right|}{\left|A\cup B\right|}=\frac{\left|A\cap B\right|}{\left|A\right|+\left|B\right|-\left|A\cap B\right|} \end{equation*}

where $A$ and $B$ are the characteristic vectors of the two drugs, $J\left(A,B\right)$ indicates the similarity between the vectors *A* and *B*, $\left|A\cap B\right|$ denotes the intersection of $A$ and $B$, and $\left|A\cup B\right|$ denotes the concatenation set.

### Graph attention network

Graph attention networks (GAT) are neural network models that introduce an attention mechanism [29]. The core idea lies in assigning a specific weight to each neighbor node, dynamically calculated based on the correlation or importance between nodes. By using these weights to aggregate the information of neighboring nodes, GAT effectively learns node representations to better capture the complex relationships in the graph structure. This approach not only enhances the model’s representation capability but also allows GAT to excel in handling graph data with different connectivity patterns. According to the original paper on GAT [30], the set of node features $X=\left\{{x}_1,\cdots \cdots, {x}_N\right\}\ {x}_i\in{R}^d$ of the graph is used as input to the GAT layer. The feature set is linearly transformed using on the node feature transformation matrix $W\in{R}^{d\times{d}^{\prime }}$, where $d$and ${d}^{\prime }$ are the dimensions of the input and output node features, respectively. Additionally, the attention weights of neighboring nodes to the central node are calculated as follows:


(2) \begin{equation*} {e}_{ij}=a\left(W{\overrightarrow{h}}_i,W{\overrightarrow{h}}_j\right) \end{equation*}

where $a\left(\cdotp \right)$ denotes the function that calculates the correlation of two nodes. To better assign weights, the correlations computed with all neighbors are uniformly normalized using the softmax function:


(3) \begin{equation*} {\alpha}_{ij}=\mathrm{softma}{\mathrm{x}}_j\left({e}_{ij}\right)=\frac{\mathit{\exp}\left({e}_{ij}\right)}{\sum_{v_b\in N\left({v}_i\right)}\mathit{\exp}\left({e}_{ib}\right)} \end{equation*}

Once the weight coefficients have been obtained, the new feature vector $\overrightarrow{x_i}$ of the node ${v}_i$ can be obtained along the lines of the weighted summation of the attention mechanism as:


(4) \begin{equation*} {\overrightarrow{x}}_i=\sigma \left({\sum}_{v_j\in N\left({v}_i\right)}{\alpha}_{ij}W{\overrightarrow{x}}_j\right) \end{equation*}

The multi-head attention mechanism introduces multiple attention heads, where the input information is divided into multiple parts, and each attention head independently learns and attends to different information. Essentially, it repeats the previous operation of calculating the attention weights multiple times.


(5) \begin{equation*} {\overrightarrow{x}}_i^{\prime }=\prod_{k=1}^K\sigma \left({\sum}_{v_j\in N\left({v}_i\right)}{\alpha}_{ij}^k{W}^k{\overrightarrow{x}}_j\right) \end{equation*}

where $K$ is the number of heads.

### Message passing neural network

A message-passing neural network (MPNN) is a distinctive neural network architecture characterized by its message-passing mechanism [31]. In an MPNN, information flows not only between neuronal nodes but is also continuously updated and transformed during the transmission process. This information transfer closely simulates the complex and precise information exchange process between neurons in biological neural networks. MPNN employs an efficient neighbor aggregation strategy, where the representation vector of a node is not isolation, but is computed from the representation vectors of its neighboring nodes through a cyclic aggregation and transfer operation. This process is essentially a message-passing process, where each node receives information from its neighboring nodes and updates its state representation accordingly, as shown in Fig. 2(b). In this way, the MPNN captures localized dependencies among nodes and constructs a more accurate and comprehensive network representation. Given an undirected graph $G$, the feature of the node ${v}_i$ at the $t-\mathrm{th}$ iteration $t$ is represented as ${x}_i^{(t)}\in{R}^d$, ${x}_i^{\left(t+1\right)}\in{R}^d$ is then updated using the following formulas.


(6) \begin{equation*} {m}_i^{\left(t+1\right)}=\sum_{v_j\in N\left({v}_i\right)}{M}_t\left({x}_i^{(t)},{x}_j^{(t)},{e}_{ij}\right) \end{equation*}

(7) \begin{equation*} {x}_i^{\left(t+1\right)}={U}_t\left({x}_i^{(t)},{m}_i^{\left(t+1\right)}\right) \end{equation*}

where ${M}_t$ and ${U}_t$ represent the message function and the node update function, respectively.

To obtain a graph-level feature ${h}_G$, readout operation integrates all the node features among the graph $G$:


(8) \begin{equation*} {h}_G=R\left({x}_i^T:{v}_i\in G\right) \end{equation*}

where $R$ denotes readout function, and $T$ is the total number of iterations.

## Development of computational methods

**Figure 3:**
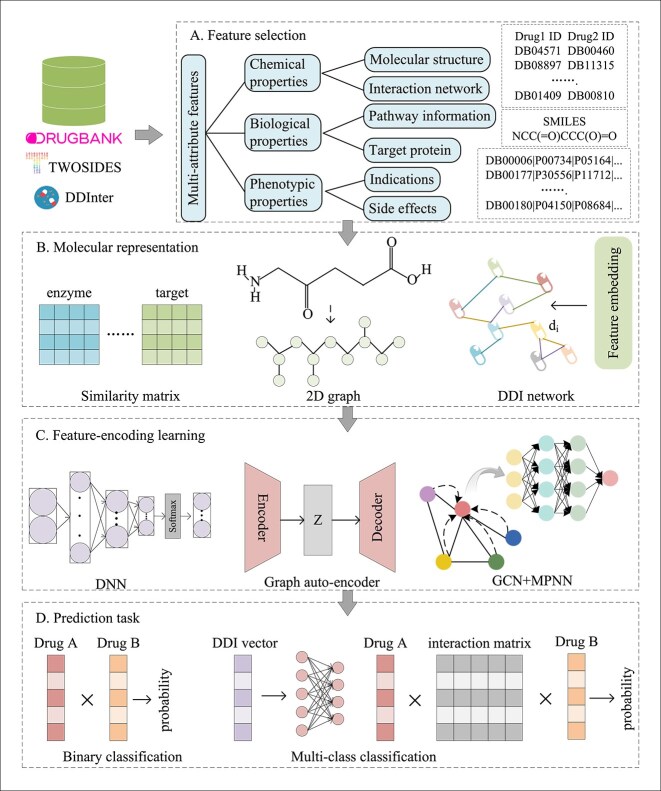
The pipeline of deep learning methods for DDI prediction. In general, drugs and their associated information (e.g*.* IDs, names, and SMILES sequences) are obtained from accessible data sources such as the DrugBank, the TWOSIDES and DDInter dataset. Various molecular representations (e.g*.* similarity matrices or 2D graphs) are then used to transform the collected drug information into a computer processable format. Then, deep network models are utilized to learn potential feature vectors. Finally, the appropriate prediction results or scores are generated based on the specific prediction task.

In recent years, researchers have explored and proposed numerous effective methods for DDI prediction in the field of deep learning. Table 3 provides detailed information on all the papers reviewed in this study. These methods can be categorized into three main types: similarity-based methods, network-based methods, and ensemble learning-based methods. This categorization helps us better understand and apply these deep learning techniques to solve real-world problems. Figure 3 illustrates a pipeline of deep methods for DDI learning.

**Table 3:** List of all papers reviewed in this study

Category	Method	Year	Drug-related information	Task	Algorithm	Code
Similarity-based approach	NMDADNN [33]	2021	Chemical substructure, Target, Enzyme, pathway, ATC code	Binary classification	Random walk+DNN	–
	MDF-SA-DDI [34]	2022	Chemical substructure, Target, Enzyme	Multi-class classification	CNN + Auto-encoders +Siamese network	Link
	DDI-IS-SL [35]	2022	Chemical substructure, Target, Enzyme, pathway	Binary classification	Semi-supervised learning	–
	DeepDDI [36]	2018	SMILES	Binary/multi-class classification	Structural similarity+DNN	Link
	CNN-Siam [37]	2023	Chemical substructure, Target, Enzyme,	Multi-class classification	CNN + Siamese network	Link
	CNN-DDI [38]	2022	Categories, Targets, Pathways, Enzymes	Multi-class classification	CNN + FC layer	–
	CASTER [39]	2020	SMILES	Binary classification	Encoder+Decoder	Link
Network-based approach	SSF-DDI [42]	2024	SMILES	Binary classification	D-MPNN+GAT+CNN + SAGP00ling	Link
	SumGNN [43]	2021	Drug ID	Multi-class classification	GNN + Attention	Link
	RANEDDI [44]	2022	Drug ID	Binary/multi-class classification	RotatE+DNN	Link
	GNN-DDI [32]	2022	SMILES	Binary classification	Graph embedding+FC layer	Link
	MIRACLE [45]	2021	SMILES	Binary classification	GCN + BAMPN+Contrastive learning	Link
	DDI-MDAE [46]	2019	Drug ID, Chemical substructure, Target, Enzyme, pathway	Binary classification	Multiple auto-encoders +DOM Forest	–
	ACDGNN [47]	2023	Drug, Diseases, Genes	Binary classification	TransE+Attention	Link
	DSN-DDI [48]	2023	SMILES	Binary classification	Encoder+Decoder	Link
	DPDDI [49]	2020	Drug ID	Binary classification	GCN + DNN	Link
	DANN-DDI [50]	2022	Drug ID	Binary classification	SDNE+Attention	Link
	SSI-DDI [51]	2021	SMILES	Binary classification	GAT+Attention	Link
	GMPNN-CS [52]	2022	SMILES	Binary classification	MLP + Linear transformer +GMPNN	Link
Integration-based approach	MDNN [1]	2021	knowledge graph, heterogeneou graph	Binary classification	Multimodal representation +GNN	–
	GoGNN [53]	2020	SMILES	Multi-class classification	GNN + Dual-attention	Link
	MSFF-MA-DDI [54]	2024	Chemical substructure, Target, Enzyme	Multi-class classification	CNN + Heterogeneous embedding	Link
	MUFFIN [55]	2021	Drug ID, SMILES	Binary/multi-class classification	MPNN+TransE+FC layer	Link
	BioDKG-DDI [56]	2022	SMILES	Binary classification	BiLSTM+Attention+DNN	–
	Multi-SBI [57]	2022	Chemical substructure, Target, Enzyme, pathway	Multi-class classification	PU-sampling+DNN	Link
	AMDE [58]	2022	SMILES	Binary classification	MPAN+Transformer	Link
	MSEDDI [59]	2023	Knowledge graph, SMILES	Multi-class classification	TransE+Self-attention +MLP	Link
	DDIMDL [1]	2020	Chemical substructure, Target, Enzyme, pathway	Multi-class classification	DNN	Link
	DGDFS [60]	2022	Chemical structure, side effect	Binary classification	Norm equality constraints	–
	MFFGNN [63]	2022	Drug ID, SMILES	Binary classification	Graph interaction networks +BiGRU+MLP	-

Note: “–” indicates that the information was not reported in the original paper.

### Similarity-based approaches

Similarity-based methods assume that similar drugs are likely to produce similar DDIs. For example, if Drug A interacts with Drug B to produce an interaction of type r, it is assumed that if Drug C is similar to Drug A, then Drug C will also interact with Drug B to produce an interaction of type r. These methods use various drug characteristics to measure similarity and identify potential interactions between drugs [32]. Currently, similarity-based methods have yielded promising results. To predict DDIs, Yan *et al.* [33] integrated five types of drug information (chemical substructure, drug-target associations, drug-enzyme associations, drug-pathway associations, and Anatomical Therapeutic Chemical (ATC) code) to obtain potential drug features for predicting DDI types. They first constructed a similarity network using Jaccard coefficients, then extracted topological similarity using a restarted random walk algorithm and positive pointwise mutual information. The five similarities were encoded and fused using a multi-modal deep autoencoder. Finally, a deep neural network was implemented on latent drug features to recognize DDIs. MDF-SA-DDI [34] processes similar drug features through four differentiated drug fusion networks, which extract potential feature vectors and perform output prediction *via* a transformer self-attention mechanism. Yan *et al.* [35] proposed a DDI prediction method based on integrated similarity and semi-supervised learning (DDI-IS-SL). DDI-IS-SL integrates chemical, biological, and phenotypic data of drugs and computes drug feature similarity using the cosine similarity method. Additionally, Gaussian interaction probability kernel similarity between drugs is calculated based on the known DDIs. Finally, the method calculates interaction likelihood scores between drug pairs using a semi-supervised learning method called the regularized least squares classifier. DeepDDI [36] uses drug names and SMILES as inputs to construct an 8-layer DNN model that is multi-labeled and outputs predicted probabilities for 86 DDI types, with interactions considered likely if the probability of a particular class exceeds a threshold of 0.47. CNN-Siam [37] employs one-hot encoding for three types of data: chemical structure, target, and enzyme, calculates drug similarity using the Jaccard function, and feeds the data into a CNN network structure. CNN-DDI [38] computes the similarity matrix of drugs based on drug classes, targets, pathways and enzymes to obtain feature vectors, which are then fed into a CNN prediction module. Huang *et al.* [39] proposed a new method called CASTER, which extracts substructures by deep traversal of SMILES, calculates the interaction probability of each substructure between drug pairs, and sums these probabilities to obtain the overall interaction probability between two drugs. As research advanced, not only were the interactions between two drugs studied, but also the specific types of chemical reactions they produced. Dang *et al.* [40] calculated similarity matrices based on two types of features, which were then fed into various classification algorithms to predict the DDI types of histamine antagonist drugs. A limitation of these approaches is that similar drugs (or chemical entities) do not necessarily exhibit the same biological activity [41]. Therefore, these methods need to account for the inherent variability in biological activities among similar chemical structures to enhance, prediction accuracy and reliability.

### Network-based approaches

The network-based approach utilizes known drug DDI information to construct a heterogeneous network, where drugs are viewed as nodes and the known relationships between them serve as edges connecting these nodes. By learning the embedded representations of the nodes in the network, we can efficiently predict potential edges, i.e*.* possible interactions between drugs. The establishment and study of drug interaction networks can help researchers better understand drug interactions and mechanisms of drug efficacy, as well as discover new therapeutic strategies and drug combination regimens. SSF-DDI [42] uses D-MPNN to extract structural information from drug 2D maps and GAT to capture important substructural information for DDI prediction. SumGNN [43] effectively utilizes knowledge graphs to assist in drug interaction prediction. Yu *et al.* [44] proposed an end-to-end learning model recognizing that different relationships have different effects on drug embeddings. To fully utilize the multi-relational information of drugs, they employed a relation-aware model to learn embedded representations, resulting in more expressive drug representations. Al-Rabeah and Lakizadeh [32] proposed a deep learning-based DDI recognition approach in two phases. In the first phase, drug information from various sources is collected and integrated by forming attribute heterogeneous networks to generate drug embedding vectors. In the second phase, these aggregated representation vectors are used for DDI prediction through a deep multi-model framework. Wang *et al.* [45] proposed a multi-view graph comparative representation learning method for DDI recognition, based on network-enhanced spurious drug interaction recognition studies, to capture both inter-view molecular structures and intra-view interactions between molecules. The method treats the drug interaction network as a graph with multiple views, where each node is itself a drug molecule graph instance. In the feature learning phase, the method encodes DDI relationships with GCNs and captures drug molecule structure information using a key-aware attention message propagation method. Wang *et al.* also proposed a new unsupervised contrastive learning component to balance and integrate multi-view information. Liu *et al.* [46] proposed a multimodal deep auto-coding method to learn a unified representation of drugs from drug feature networks. ACDGNN [47] combines gene and disease information to construct a heterogeneous network, reducing heterogeneity between different types of entities through cross-domain transformation operations. DSN-DDI [48] employs an MPNN-based feature learning module to learn drug substructures from both single-drug (intra-view) and drug-pair (inter-view) views, achieving good prediction results. DPDDI [49] effectively captures the topological relationships between drugs in the DDI network using GCN, learing the potential features of the drugs, and concatenates these potential feature vectors to form a comprehensive feature representation for accurate DDI prediction. DANN-DDI [50] forms a drug feature network and learns drug embeddings using a graph representation learning method, then inputs the representation of drug pairs into a deep neural network to predict potential DDIs. Both SSI-DDI [51] and GMPNN-CS [52], leverage deep learning’s superior feature extraction capabilities. SSI-DDI utilizes GATs [30] to learn and integrate comprehensive feature representations of drugs from substructures, while GMPNN-CS focuses on capturing information about chemical substructures of different sizes and shapes from molecular graphical representations through gated message-passing neural networks. These methods not only improve prediction accuracy but also deepen our understanding of the relationship between drug molecular structures and their interactions. MIRACLE [45] employs comparative representation learning of multi-view graphs, capturing both structural information within molecules and relational information about intermolecular interactions, and balancing features learned from different views using comparative learning to obtain more accurate drug representation vectors. While these advanced network-based approaches are highly effective, they still face challenges. Continuous refinement is needed to better capture the complexity and variability of drug interactions. Integrating more diverse and high-quality data sources could further enhance the predictive power and reliability of these models. Additionally, exploring new machine-learning techniques and hybrid models may provide deeper insights into drug interactions and lead to the discovery of novel therapeutic strategies [28].

### Integration-based approaches

Despite significant advances in previous methods, improving prediction accuracy remains crucial. Integration-based approaches effectively fuse two or more different types of feature information efficiently to enhance the accuracy and reliability of predictions. For example, MDNN [1] leverages the complementary nature of multimodal data by designing a dual-pathway framework to handle drug knowledge graphs and heterogeneous features separately. GoGNN [53] comprehensively extracts drug features from two perspectives: intra-entity and inter-entity interactions. MSFF-MA-DDI [54] captures global features of molecular sequences through a self-attention layer based on positionally encoded embedded blocks, encodes chemical sub-structures, target, and enzyme features using self-encoders, and performs multi-source feature fusion using multi-head attention. MUFFIN [55] jointly learns drug representations based on the structural information of the drug itself and a biological knowledge graph with rich medical information, alleviating the constraints of limited labeling data on deep learning models. BioDKG-DDI [56] skillfully fuses molecular structural features, global association characterization in the Drug Knowledge Graph (DKG), and drug-functional similarity features through attention mechanism to predict novel DDIs. Huang *et al.* [57] encoded 1D sequential information and heterogeneous networks separately, using PU-sampling to select negative samples with high confidence and reduce sampling noise. AMDE [58] designed 2D graph feature encoder and one-dimensional (1D) sequence feature encoder modules to encode 2D graph features and 1D SMILES sequences using message-passing attention networks and Transformers, respectively. MSEDDI [59] designed a three-channel network to handle knowledge graph embeddings based on biomedical networks, symbolic embeddings based on SMILES sequences, and chemical structure embeddings based on molecular graphs. Deng *et al.* [1] designed a multimodal deep learning framework that enhances DDI prediction by integrating multiple drug features. Zhu *et al.* [60] selected eight key attributes and developed a discriminative learning algorithm to learn the attribute representations of drug pairs causing adverse reactions. This algorithm effectively utilized the consensus information and complementary advantages of these attributes to predict multi-attribute adverse drug–drug interactions (ADDI). Addition, they crafted three dependency bootstrap terms to reveal potential associations between molecular structure, side effects, and ADDI, guiding the feature selection process. Ultimately, they proposed a discriminative feature selection model, DGDFS, which significantly improved ADDI prediction accuracy. He *et al.* [61] proposed a multi-type feature fusion method to fully utilize the topological information of drugs, SMILES sequences and drug interaction network information. A gating mechanism was introduced at the feature fusion stage to address the over-smoothing problem, resulting in improved performance. In DDI prediction, the fusion of multiple features is critical due to the complexity of drug molecular structures, the diversity of biological activities, and the subtlety of DDIs. Therefore, designing a reasonable feature fusion strategy to fully leverage the advantages of various features and enhance the accuracy and stability of DDI prediction is a significant challenge for integrated methods.

### Challenges and opportunities

Although a large number of studies have focused on predicting DDIs with high accuracy, challenges remain and promising future directions are as follows.

#### Data sparsity and imbalance

Many datasets contain limited information on known DDIs, meaning that only a small fraction of all possible drug pairs have documented interactions. This scarcity makes it challenging to build comprehensive models capable of accurately predicting interactions across a wide range of drugs. Additionally, these datasets often lack validated drug pairs confirmed to have no interactions. The absence of such negative samples, introduces significant data imbalance. Most datasets are heavily skewed toward positive interactions, leading to biased models that may overestimate the likelihood of interactions [62]. This imbalance can result in models generating a high number of false positives, predicting interactions where there are none, thereby reducing prediction reliability.

To address these issues, it is crucial to develop strategies for constructing more balanced and comprehensive datasets. This includes actively seeking and validating negative samples to ensure a more representative distribution of drug pairs. Advanced data augmentation techniques and synthetic data generation methods can also mitigate the effects of data sparsity and imbalance. By improving the quality and balance of datasets, we can enhance the accuracy and reliability of DDI prediction models, bringing them closer to real-world applicability.

#### Interpretability and generalization of prediction models

Many existing deep learning and graph learning models hidden representations of the data, primarily capture implicit correlations within the data through latent embeddings and hidden representations [45, 57, 63]. While these embeddings can be effective for making predictions, they often fail to provide clear, understandable explanations for why certain drug interactions are predicted. This lack of transparency makes it difficult for researchers and clinicians to trust and validate the model’s outputs. Improving interpretability involves developing methods that make the internal workings of the models more transparent [64]. Techniques such as attention mechanisms, feature importance scores, and visualization tools can help elucidate how the model reaches its conclusions. For instance, highlighting which features were most influential in predicting a particular interaction can provide valuable insights into the underlying biological processes.

Moreover, prediction models must demonstrate strong generalization abilities. They should perform consistently well across various datasets and real-world conditions, not just within the specific contexts they were trained in [65]. Drug interaction data can vary widely in terms of quality, source, and scope. Therefore, models need to be robust enough to handle these variations and still deliver reliable predictions. This is particularly important in clinical settings where diverse patient populations and treatment protocols are encountered. Enhancing generalization requires training models on diverse and comprehensive datasets to capture a wide range of interaction patterns. Cross-validation and rigorous testing on independent datasets are essential steps in this process [66]. Additionally, incorporating domain knowledge and leveraging transfer learning techniques can help models adapt to new and unseen data more effectively. By addressing these aspects, we can develop DDI prediction models that are not only accurate but also interpretable and generalizable, making them more practical and reliable for real-world applications.

## Conclusions

In this study, we systematically review various deep learning applications in drug interaction prediction. We categorize these methods into similarity-based, network-based, and integration-based approaches. We present data sources, and summarize widely used molecular representations, as well as common models for constructing similarity matrices and extracting features from graph data. This categorization outlines the development of computational methods in recent years.

Although significant research has been focused on predicting DDIs and achieving high predictive performance, several challenges and promising future directions remain. Challenges include data sparsity and imbalance, the complexity and diversity of drug interactions, and the interpretability and generalization abilities of prediction models. Future directions involve further integrating multi-source information, developing more advanced algorithms and models, and exploring the application of DDIs in personalized medicine and precision drug use. Through continuous research and innovation, we expect to overcome existing challenges and advance DDI prediction technology.

**Key Points**

- This review categorizes recent approaches in drug–drug interaction (DDI) prediction into similarity-based, network-based, and integration-based methods, providing a comprehensive overview of the current landscape.
- The article highlights the most commonly used databases and molecular representations, offering readers a clear understanding of the foundational data and representations employed in DDI prediction studies.
- It details several widely used models for extracting drug features, enhancing the reader’s knowledge of the tools and techniques employed in the field.
- This paper presents an intuitive and easy-to-understand research framework by presenting the basic process of DDI prediction in the form of a diagram. It discusses the limitations of existing methods and suggests promising future research directions, helping readers grasp the core steps, methods, and potential advances in DDI prediction.

## Notes

**Yan Xia** is currently a PhD candidate in Electronic Information at Hainan University, Haikou, China. Her research interests include bioinformatics, machine learning and deep learning.

**An Xiong** is currently a master's student in Software Engineering at Hainan University, Haikou, China. Her research interests include bioinformatics and deep learning.

**Zilong Zhang** is currently an associate professor in the School of Computer Science and Technology, Hainan University. He received the Ph.D. degree in bioinformatics from the University of Tokyo, Japan. His research interests include bioinformatics, machine learning and graph neural network.

**Quan Zou** is currently a Professor in the Institute of Fundamental and Frontier Sciences, University of Electronic Science and Technology of China, Chengdu, China. He received the PhD degrees in computer science from Harbin Institute of Technology, China. His research is in the areas of bioinformatics, machine learning and parallel computing.

**Feifei Cui** is currently an associate professor in the School of Computer Science and Technology, Hainan University. She received the Ph.D. degree in bioinformatics from the University of Tokyo, Japan. Her research interests include bioinformatics, deep learning and biological data mining.
